# Unveiling disulfidptosis-linked lncRNA signatures: insights into the immune microenvironment and drug responsiveness in oral squamous cell carcinoma

**DOI:** 10.3389/fgene.2025.1650544

**Published:** 2025-11-10

**Authors:** Xue Meng, Lichao Cao, Yantao Liu, Sihan Wang, Weixian Liu, Guangping Zhang, Siqi Guo, Fangyu Qi, Tingting Wang, Yuhe Xia, Ying Ba, Hezi Zhang, Lijun Fang

**Affiliations:** 1 Department of Oral and Maxillofacial Surgery, Shengjing Hospital of China Medical University, Shenyang, China; 2 Department of Research and Development, Shenzhen Nucleus Gene Technology Co., Ltd., Shenzhen, China

**Keywords:** oral squamous cell carcinoma, disulfidptosis, machine learning, drug, lncRNA, immune

## Abstract

**Objectives:**

Oral squamous cell carcinoma (OSCC) has a highly incidence rate and mortality rate all over the world. Hitherto, there are limited studies on survival significance between disulfidptosis-related lncRNAs (DRLs) and OSCC. Therefore, this study was conducted to investigate the potential role of these DRLs and provide some theoretical support in the clinical treatment of OSCC.

**Methods:**

OSCC-related lncRNAs and disulfidptosis-related genes (DRGs) were retrieved from public databases. Using Pearson correlation, machine learning, and expression profiling, we identified differentially expressed DRLs (DE-DRLs), developed a DE-DRLs-based risk model and independent prognostic nomogram, performed immunological and tumor microenvironment analyses to explore DE-DRLs regulatory mechanisms, predicted potential drugs for OSCC, and validated bioinformatics findings.

**Results:**

In this study, 9 DE-DRLs were identified that correlated with OSCC. The risk model and nomogram showed good clinical utility for assessing the likelihood of OSCC occurrence. Patients exhibiting elevated levels of eosinophils, activated natural killer (NK) cells, or naïve CD4^+^ T cells experienced significantly poorer overall survival (OS), and patients with high tumor mutational burden (TMB) had worse prognosis. 12 drugs were identified for OSCC treatment, such as BMS-754807_2171 and Foretinib_2040.

**Conclusion:**

Our study identified 9 DE-DRLs correlated with OSCC, which will be a personalized prediction tool for prognosis and immune responses in OSCC patients.

## Graphical Abstract

**Figure:**
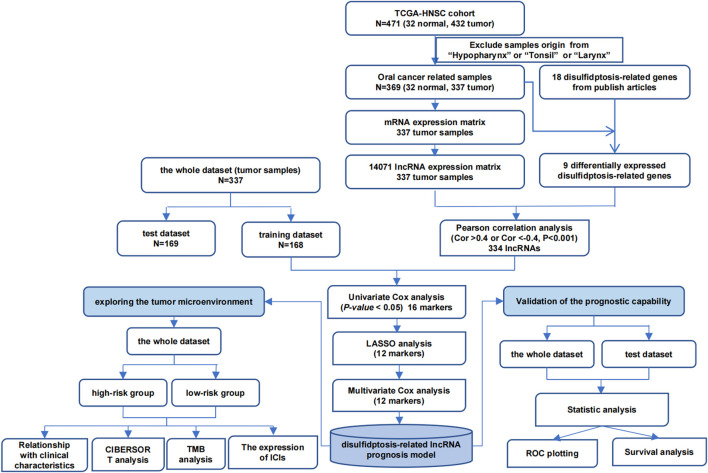

## 1 Introduction

Oral squamous cell carcinoma (OSCC) is a common heterogeneous oral malignancy (Wang. et al., 2021a). Smoking, excessive alcohol consumption, betel quid chewing, and human papilloma virus are risk factors for OSCC (Nokovitch et al., 2023). Approximately 600,000 patients are deeply troubled by OSCC each year, representing about 4% of all tumors (Pekarek et al., 2023). Oral cavity provides convenience for clinical examination, however the diagnosis of OSCC is in advanced stages due to misdiagnosis by the doctor or ignorance by patients (Jagadeesan et al., 2024). Meantime, the prognosis is unsatisfactory for patients with OSCC, the 5-year survival rate is as low as 40%–50% (Chai et al., 2020). Currently, the primary treatment modalities for OSCC include surgery, chemotherapy, and novel cellular therapies. While these are the mainstay of clinical management, their therapeutic efficacy remains suboptimal due to strong side effects, high costs of novel cellular therapies, and the propensity of OSCC cells (Jagadeesan et al., 2024). Therefore, there is a pressing need to increase survival rates and quality of life of OSCC patients by developing new and reliable prognostic evaluation method.

Disulfidptosis is a novel type of regulated cell death, which is associated with metabolic changes and has a strong effect on anti-tumor immune response (Liu et al., 2023). In addition, the metabolism of disulfides in cancer cells is connected with immune evasion, metastasis, and resistance of tumor cells (Shrihari et al., 2022; Wang et al., 2021b; Zhang et al., 2022a). As the source of programmed cell death, disulfidptosis is expected to provide a new approach to cancer therapy. So far, the role of disulfidptosis in OSCC is still quite lacking. Meanwhile, long non-coding RNAs (lncRNAs) are critical to the tumorigenesis and progression, and can be considered key factor to promotion and suppression of tumor due to their dysregulation in cancer (Ahmad et al., 2023; Kitajima et al., 2023). Currently, studies on the cell death patterns of OSCC have found that ferroptosis-related lncRNAs (Qiu et al., 2022), cuproptosis-related lncRNAs (Gong et al., 2024), and pyroptosis-related lncRNAs (Xin et al., 2022) are crucial prognostic biomarkers and therapeutic targets for OSCC. However, there is a lack of research on the role of disulfidptosis-related lncRNAs (DE-DRLs) in OSCC.

Here, we systematically structured a dependable DE-DRLs for predicting prognosis of OSCC, and explored the relationship between the prognostic model and clinicopathological information and the tumor immune landscape, and investigate the role of key DE-DRLs in OSCC. The implications of our findings are expected to generate guidance for tailoring personalized treatment strategies, and provide a foundation for further research on the mechanism of disulfidptosis in OSCC.

## 2 Acquisition of data

The lncRNAs sequencing information, which exclude samples origin from “hypopharynx”, “tonsil” or “larynx” of the TCGA-HNSC were acquired from the Cancer Genome Atlas (TCGA) database (https://portal.gdc.cancer.gov/), in which 14,071 lncRNAs were identified for the further analysis. The TCGA-HNSC comprised 337 tumor samples along with 32 control samples, samples that were not representative of the oropharyngeal region and that did not affect the prognosis of HPV were excluded, the clinical pathological information was shown in Supplementary Table S1. Additionally, 18 DRGs were obtained from the literature (Dong et al., 2023).

## 3 Statistical methods

### 3.1 Identification of DE-DRLs

Firstly, the DE-DRGs were analyzed utilizing Wilcoxon test between tumor and control group in TCGA-HNSC (P < 0.05). Among the 337 tumor samples in TCGA-HNSC, a Pearson correlation was constructed to illustrate the obtain DE-DRLs between the DE-DRGs and 14,071 lncRNAs (|correlation coefficient (cor)|>0.4, P < 0.05). Following this, 337 tumor samples were divided into 168 training dataset and 169 test dataset (1 : 1), the univariate Cox regression analysis of signature genes 1 was performed utilizing the survival (v 3.5.3) package (Therneau and Lumley, 2015) (P < 0.05). Moreover, the LASSO analysis with penalty parameters (lambda), β coefficient ≠ 0 and the 10-fold cross-validation was performed on signature genes 1 by glmnet (v 4.1.4) package (Friedman et al., 2021) to further screen signature genes 2. Lastly, multivariate Cox regression analysis and Wilcoxon test were utilized to obtain DE-DRLs (P < 0.05).

### 3.2 Prognostic modeling and assessment

Furthermore, independent prognostic factors and their coefficients were denoted through multivariate Cox regression assessment before calculating individual case risk scoring, using:

$$Risk\;scoring=\sum \text{Cox}coefi{\;of\;lnRNA\;\chi }^{i}\times Scaled\;expression\;value\;of\;lncRNA\;\chi i$$

Where χi represented the expression level of DE-DRLs, and coefi denoted the risk coefficient of the corresponding gene. Subsequently, the GGally (v2.3.0) package was utilized to conduct a correlation analysis on DE-DRLs and the risk coefficients of these DE-DRLs were obtained through multivariate regression analysis. Following the optimal cut-point method described by Mallardo et al. (2024), 337 brain tumor samples were divided into high- and low-risk groups based on the risk scores of DE-DRLs. Following this, the risk model was evaluated by plotting the receiver operating characteristic (ROC) curves with the utilize of timeROC (v 1.0.3) package (Blanche and Blanche, 2019) for the reliability of model (area under curve (AUC) > 0.6) and utilized the survminer (v 0.4.9) package, overall survival (OS) endpoints across groups were comparatively analyzed based on Kaplan–Meier curves in TCGA-HNSC tumor samples and test dataset (hazard ratios (HR)≠1, 95% confidence intervals (CI)).

### 3.3 Construction of nomogram

Among the 337 tumor samples, the Wilcoxon test (P < 0.05) and multifactor Cox regression analysis (survival (v 3.5.3) package) (HR ≠ 1 and P < 0.05) were conducted to obtain the independent prognostic factors of OSCC patients based on the risk score and 3 clinical characteristics (age, gender, and stage) (P < 0.05). After that, a nomogram was constructed based on the independent prognostic factors to predict mortality in patients with OSCC by rms (v 6.5.0) package (Harrell Jr et al., 2017). Additionally, the calibration curves by PredictABEL (v 1.2.4) package (Kundu et al., 2020) were also plotted to evaluate the accuracy of the predicted probabilities of the nomogram.

### 3.4 Immune microenvironment analysis

Normalized gene expression matrices, in conjunction with the CIBERSORT algorithm, were utilized to estimate the proportions of all 22 immune cell types between high - risk and low - risk group (Newman et al., 2015). Kaplan-Meier curves were then generated to assess the association of each significantly differentially abundant immune cell type (P < 0.05) with OS. Correlation analysis was utilized to demonstrate the correlation between DE-DRLs and different immune cells. The estimate (v 1.0.13) package (Yoshihara et al., 2016) was utilized to analysis the difference between high - risk and low - risk group in ESTIMATEScore, ImmuneScore, and StromalScore. The mutation profiles of the two groups were visualized using the maftools package in R on the entire dataset, and differences in TMB were evaluated via unpaired t - tests.

### 3.5 Analysis of chemotherapeutic drug sensitivity and immune checkpoints

The oncoPredict (v 0.1) package was employed to predict the half maximal inhibitory concentration (IC_50_) values for cancer drug response associated with OSCC treatment in each sample (IC_50_ < 5). Subsequently, the constructed prognostic risk score model was utilized to comparatively analyze the differences in the response to these selected drugs between the high - risk and low - risk groups. Moreover, the Wilcoxon test allowed transcriptomic expression profiles for immune checkpoints together with linked ligands across high-together with low-risk group for comparative analyses (P < 0.05). Lastly, correlation analysis was utilized to demonstrate the correlation between DE-DRLs and immune checkpoints.

### 3.6 Expression analysis of DE-DRLs

Further analysis was conducted to verify the expression of biomarkers through RT-qPCR. The OSCC tumor samples were gained from the 10 patients in Shengjing Hospital of China Medical University. And the adjacent tumor sample obtained from 4 healthy individuals were utilized as control samples. This study was approved by Ethics Committee of Shengjing Hospital of China Medical University (No. 2024PS802K). All individuals had signed an informed consent form. Total RNA of each sample was separately extracted using TRIzol (TIANGEN, Beijing, CHINA) according to the manufacturer’s guidance. Reverse transcription of total RNA to cDNA was carried out by using Hifair® Ⅲ 1st Strand cDNA Synthesis SuperMix for qPCR (gDNA digester plus) (Yisheng, Shanghai, China) based on the manufacturer’s instructions. RT-qPCR was performed utilizing the Hieff® qPCR SYBR Green Master Mix (Yisheng, Shanghai, China). The primer sequences for PCR were shown in Supplementary Table S2. GAPDH was an internal reference gene. The 2^−ΔΔCT^ method (Livak and Schmittgen, 2001) was utilized to calculate the expression of biomarkers.

## 4 Results

### 4.1 Construction of a prognostic DE-DRLs in OSCC

**FIGURE 1:**
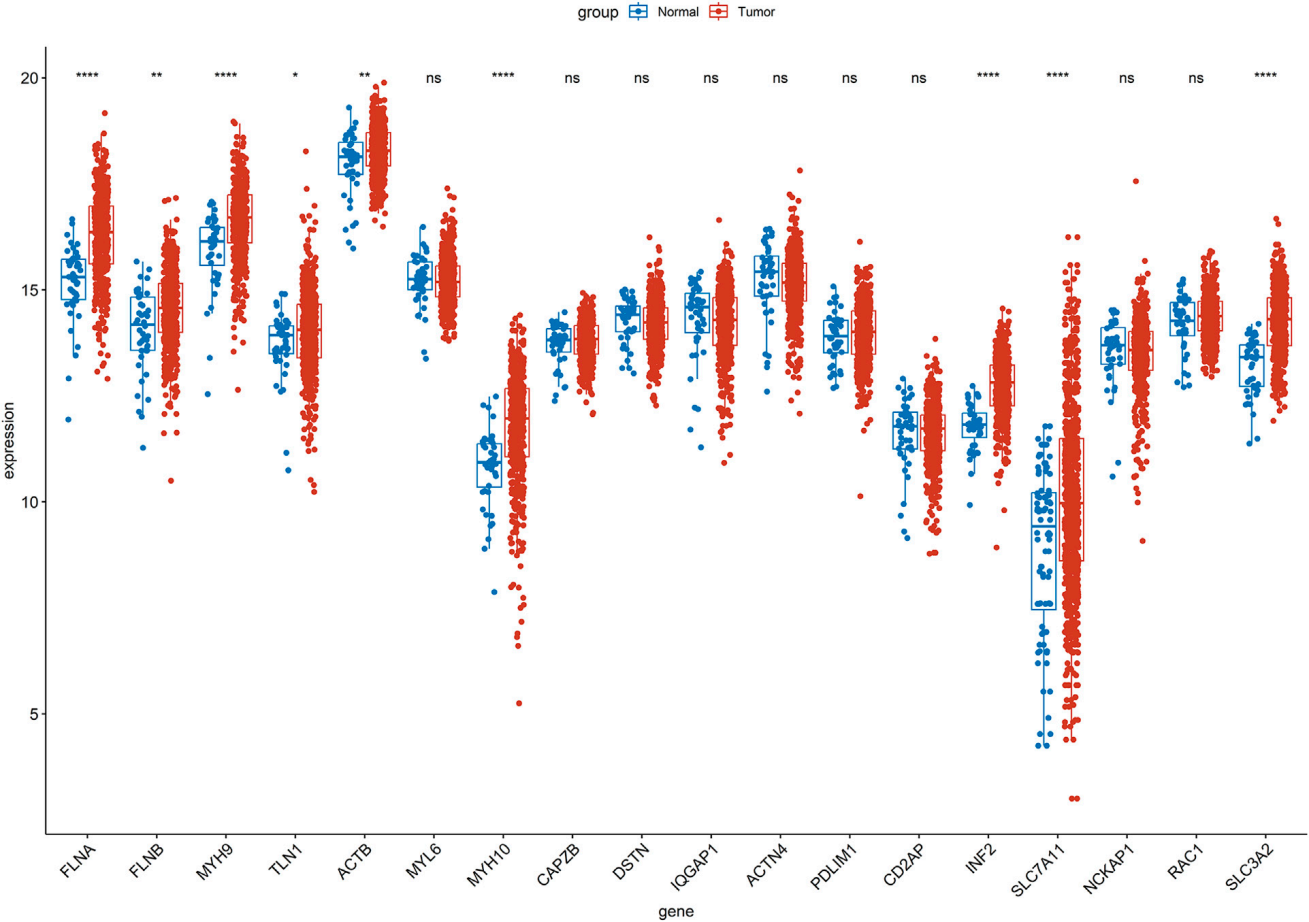
Wilcoxon test of 18 disulfidptosis-related genes (DRGs) between tumor and control group in TCGA-HNSC. “ns” indicates p > 0.05; “*” indicates p < 0.05; “**” indicates p < 0.01; “***” indicates p < 0.001; and “****” indicates p < 0.0001.

**FIGURE 2:**
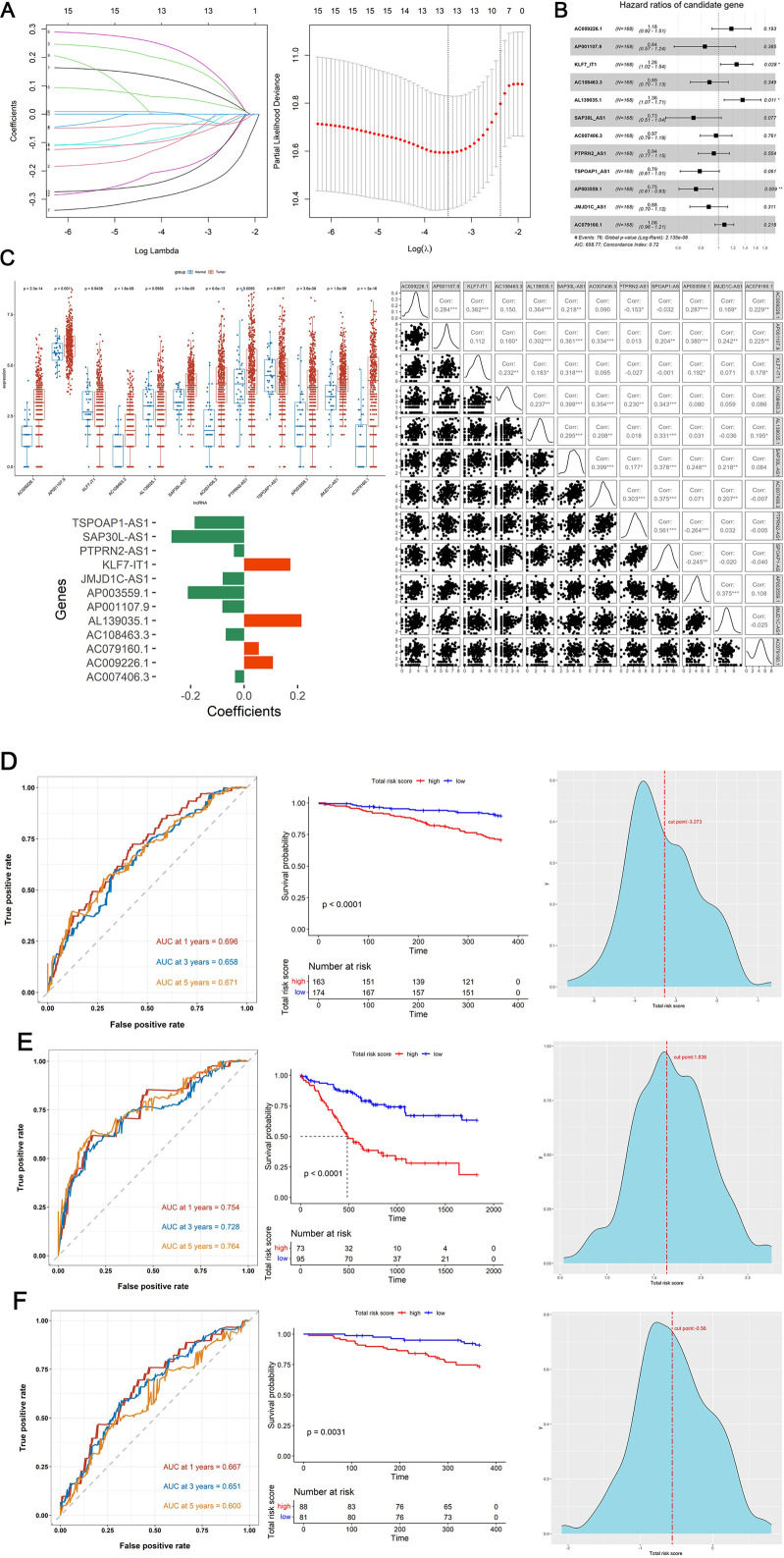
Construction and validation of prognostic model. **(A)** The variation characteristics of the coefficient of variables and the selection process of the optimum value of the parameter λ in the Lasso regression model by cross-validation method. **(B)** Multivariate Cox regression analysis of DE-DRLs. **(C)** Wilcoxon test and Pearson correlation analysis of 12 DE-DRLs (P < 0.05). (D–F) Receiver operating characteristic (ROC) curves and Kaplan-Meier (K–M) survival analysis, cut-point value in TCGA-HNSC, training and test dataset.

Firstly, the Wilcoxon test demonstrated 9 DE-DRGs significant difference between tumor and control group (Figure 1). A total of 334 DE-DRLs were identified based on Pearson correlation analysis with |coefficient |> 0.4 and P < 0.001 (Dong et al., 2023). Subsequently, we utilized univariate Cox analysis to screen out 16 signature genes 1 with prognostic significance in TCGA-HNSC (Table 1). Furthermore, 9 DE-DRLs with the optimal prognosis were ultimately identified through LASSO analysis, the multivariate Cox analysis, and Wilcoxon test (Figures 2A–C, P > 0.05), which included AC009226.1, AP001107.9, AC108463.3, SAP30L_AS1, AC007406.3, PTPRN2_AS1, AP003559.1, JMJD1C_AS1, and AC079160.1. Notably, they were all high expressed in OSCC samples (P < 0.05). The assigned a risk score to each sample by the given equation: Risk Score = AC009226.1 * 0.16323 + AP001107.9 * (−0.17272) + AC108463.3 * (−0.11501) + SAP30L_AS1 * −0.31715 + AC007406.3 * (−0.03145) + PTPRN2_AS1 * (−0.05957) + AP003559.1 * (−0.28549) + JMJD1C_AS1 * (−0.12380) + AC079160.1 * 0.07491. Pearson correlation analysis revealed that positive correlations existed among the majority of DE-DRLs. Specifically, KLF7_IT1 and AL139035.1 exhibited relatively large positive coefficients, which implies their positive correlation with the outcome within the model. In contrast, TSPOAP1_AS1 and SAP30L_AS1 showed negative or near-zero coefficients, indicating a negative or weak association with the outcome (Figure 2C). ROC curve analysis showed the DE-DRLs had a promising predictive value in the in TCGA-HNSC (1-year AUC = 0.696, 3-year AUC = 0.658, 5-year AUC = 0.671, Figure 2D). In addition, Kaplan-Meier analysis demonstrated a significantly worse prognosis in the high-risk group in contrast to the low-risk (cut-point = −3.273, P *<* 0.0001, Figure 2D). Consistent with these findings, the DE-DRLs were validated in training and test dataset (Figures 2E,F).

**TABLE 1:** Univariate Cox analysis showing the 16 prognostic DRlncRNAs.

uni_cox_sig_genes	HR	HR.95L	HR.95H	*P*.value
AC009226.1	1.216,731	1.005823	1.471,863	0.04341
AP001107.9	0.722,089	0.526,705	0.989,953	0.043105
KLF7-IT1	1.227,341	1.019247	1.47792	0.030689
AC104083.1	0.842,821	0.710,839	0.999,308	0.049076
AC108463.3	0.814,064	0.667,029	0.99351	0.042967
AL139035.1	1.204,356	1.001837	1.447,813	0.04776
SAP30L-AS1	0.657,227	0.498,327	0.866,796	0.002956
AC007406.3	0.799,589	0.678,707	0.942,002	0.007486
AC093278.2	0.802,042	0.660,801	0.973,472	0.025615
AC107959.1	0.721,176	0.557,822	0.932,366	0.012618
PTPRN2-AS1	0.84607	0.729,072	0.981,844	0.027718
TSPOAP1-AS1	0.800,445	0.676,088	0.947,676	0.009771
AP003559.1	0.835,448	0.711,548	0.980,923	0.028154
JMJD1C-AS1	0.78333	0.632,153	0.970,659	0.025603
AC079160.1	1.135,005	1.016611	1.267,187	0.024255
LINC02561	1.140,206	1.003612	1.295,391	0.043869

### 4.2 Assessment of the relationship between DE-DRLs and clinicopathological features in OSCC

**FIGURE 3:**
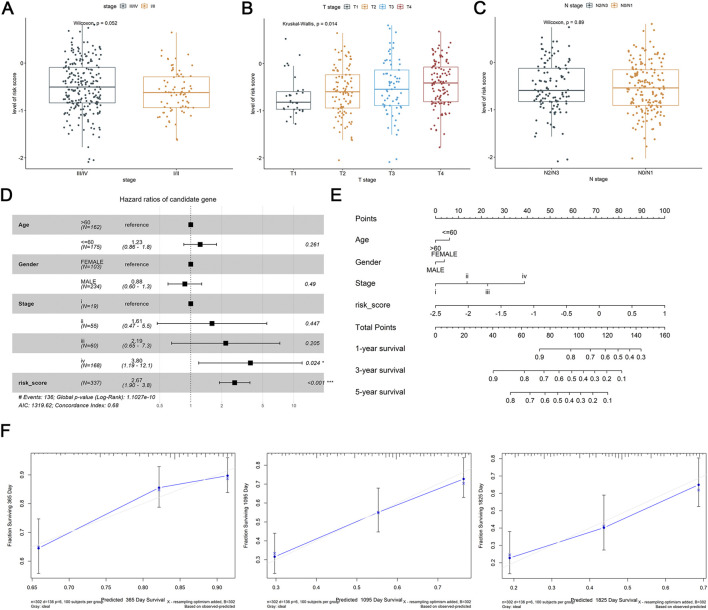
Differential expression analysis of clinicopathological features and construction and validation of nomogram. **(A–C)** Association of risk score with stage, T stage, N stage. **(D)** Multivariate Cox analysis of independent prognostic factors. **(E)** Nomogram of risk score and independent prognostic factors. **(F)** Calibration curves of nomogram at 1, 3 and 5 years.

In TCGA-HNSC tumor samples, the association of DE-DRLs with clinicopathological features were further analyzed. DE-DRLs was significantly related to T stage of OSCC (P = 0.014, Figure 3B), but not with the pathological stage (P = 0.052, Figure 3A) and N stage (P = 0.89, Figure 3C). Moreover, the multivariate Cox analysis demonstrated the risk score (HR = 2.67, 95% CI 1.80–3.80) was an independent prognostic factor for OS in OSCC patients (Figure 3D). Combination with risk scores and other clinicopathological parameters, a new nomogram were developed to make a more comprehensive prediction of patient survival at 1, 3, and 5 years (Figure 3E). Calibration curves exhibited a good degree of concordance between the actual outcomes and predicted survival probabilities for 1-, 3-, and 5-year survival rates (Figure 3F).

### 4.3 Analysis of the association between immune infiltration and DE-DRLs

**FIGURE 4:**
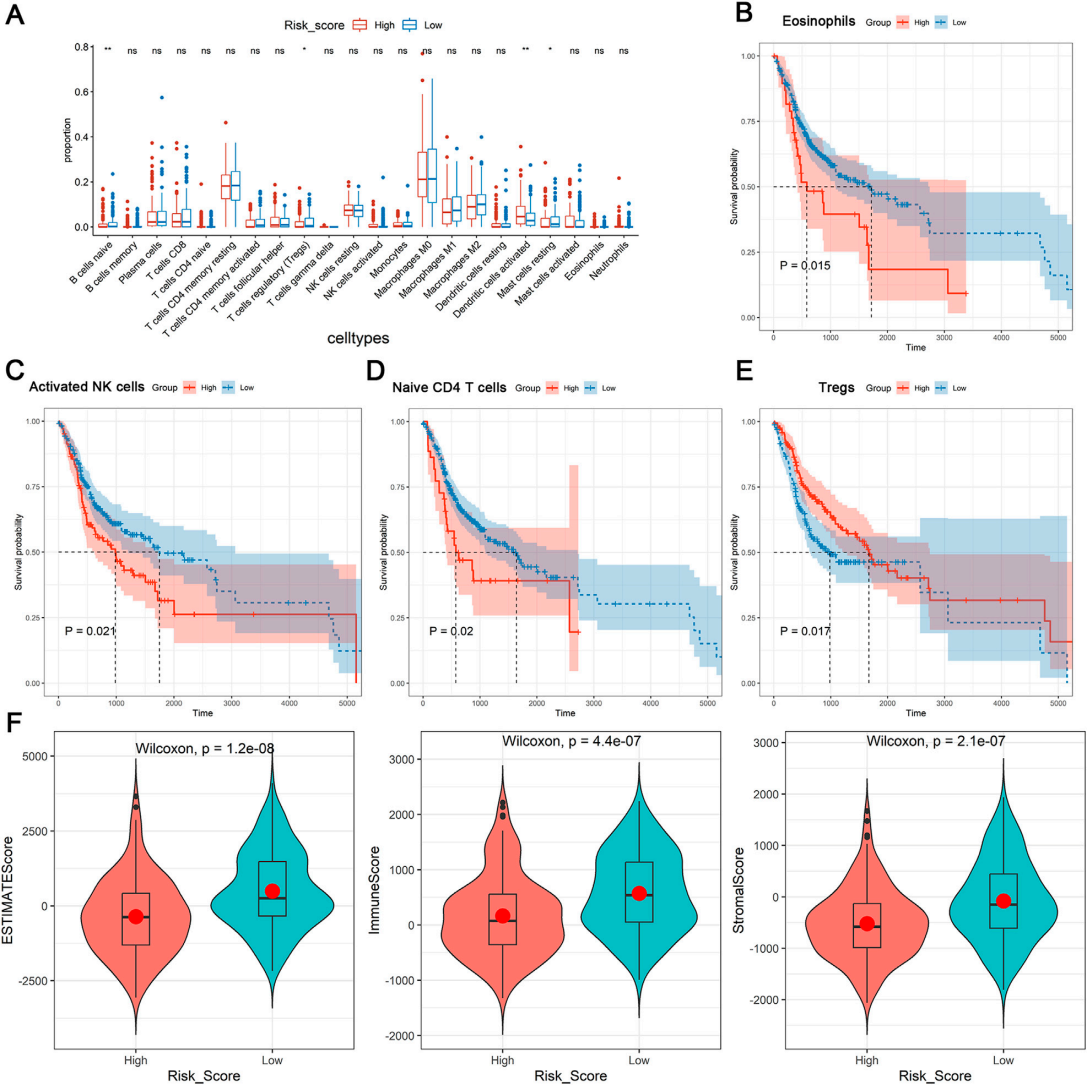
Immune microenvironment analysis. **(A)** Differences expression in infiltration levels. **(B–E)** K-M survival analysis in eosinophils, activated NK cells, naïve CD4 T cells, and Tregs between high- and low-risk groups. **(F)** Differences expression in ESTIMATEScore, ImmuneScore, and StromalScore. “ns” indicates p > 0.05; “*” indicates p < 0.05; “**” indicates p < 0.01; “***” indicates p < 0.001; and “****” indicates p < 0.0001.

After that, we further investigated the relationship between tumor microenvironment and DE-DRLs in OSCC patients of TCGA-HNSC. Significant discrepancies between the two groups were observed in the proportion of immune cells including naïve B cells, mast cells, regulatory T cells (Tregs), activated dendritic cells and resting mast cells (Figure 4A). Kaplan-Meier analysis revealed patients with high levels of eosinophils (P = 0.015), activated NK cells (P = 0.021) or naïve CD4 T cells (P = 0.020) had significantly worse OS (Figures 4B–D), whereas patients with a low level of Tregs (P = 0.017) had poor OS (Figure 4E). Overall, the DE-DRLs could reflect the immune microenvironment of OSCC patients, furthermore, DE-DRLs were generally negatively correlated with the majority of the differentiating immune cells (Supplementary Table S3). The box plot results show that significant difference were observed between high - risk and low - risk group in ESTIMATEScore, ImmuneScore, and StromalScore, which all highly expressed in low - risk group (P *<* 0.0001, Figure 4F).

### 4.4 Evaluation of the relationship between mutation profiles and DE-DRLs

**FIGURE 5:**
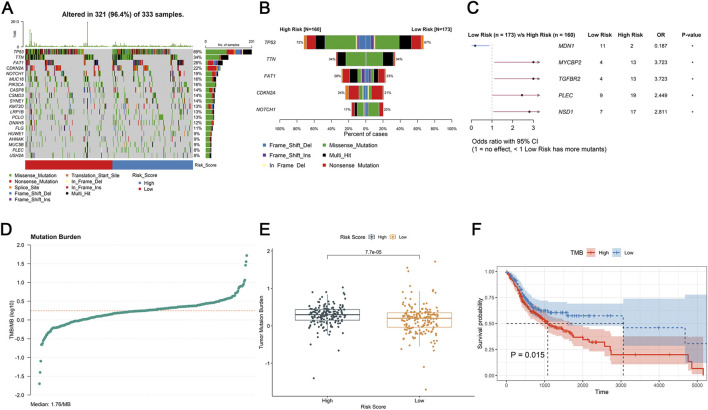
Tumor microenvironment analysis. **(A,B)** The mutational status of TMB samples. **(C)** The odds ratio of mutated genes between the high- and low-risk group in forest plot. **(D)** Mutation burder curve of OSCC tumor samples. **(E)** Differences expression in mutation burder between high- and low-risk groups. **(F)** K-M survival analysis of TMB between the high- and low-risk group.

TMB can be utilized to predict patient’s sensitivity to tumor immunotherapy. In this study, the TP53 gene mutation was highest in the TCGA-HNSC tumor samples (69%) and in the high-risk group (72%) and low-risk group (67%) (Figures 5A,B). Subsequently, as showed in Figure 5C, MDN1 (OR = 0.187) had a high mutation rate in the low-risk group, while high-risk group had high mutation rates of MYCBP2 (OR = 3.723), TGFBR2 (OR = 3.723), PLEC (OR = 2.449), and NSB1 (OR = 2.811). Moreover, the TMB of each patients (Median: 1.76/MB, Figure 5D) were calculated. There was significant difference of TMB value between the high-and low-risk group (P = 7.7e-05, Figure 5E). Meantime, patients with high TMB had worse prognosis than those with low TMB (P = 0.015, Figure 5F).

### 4.5 Prediction of drug sensitivity and chemotherapy response

**FIGURE 6:**
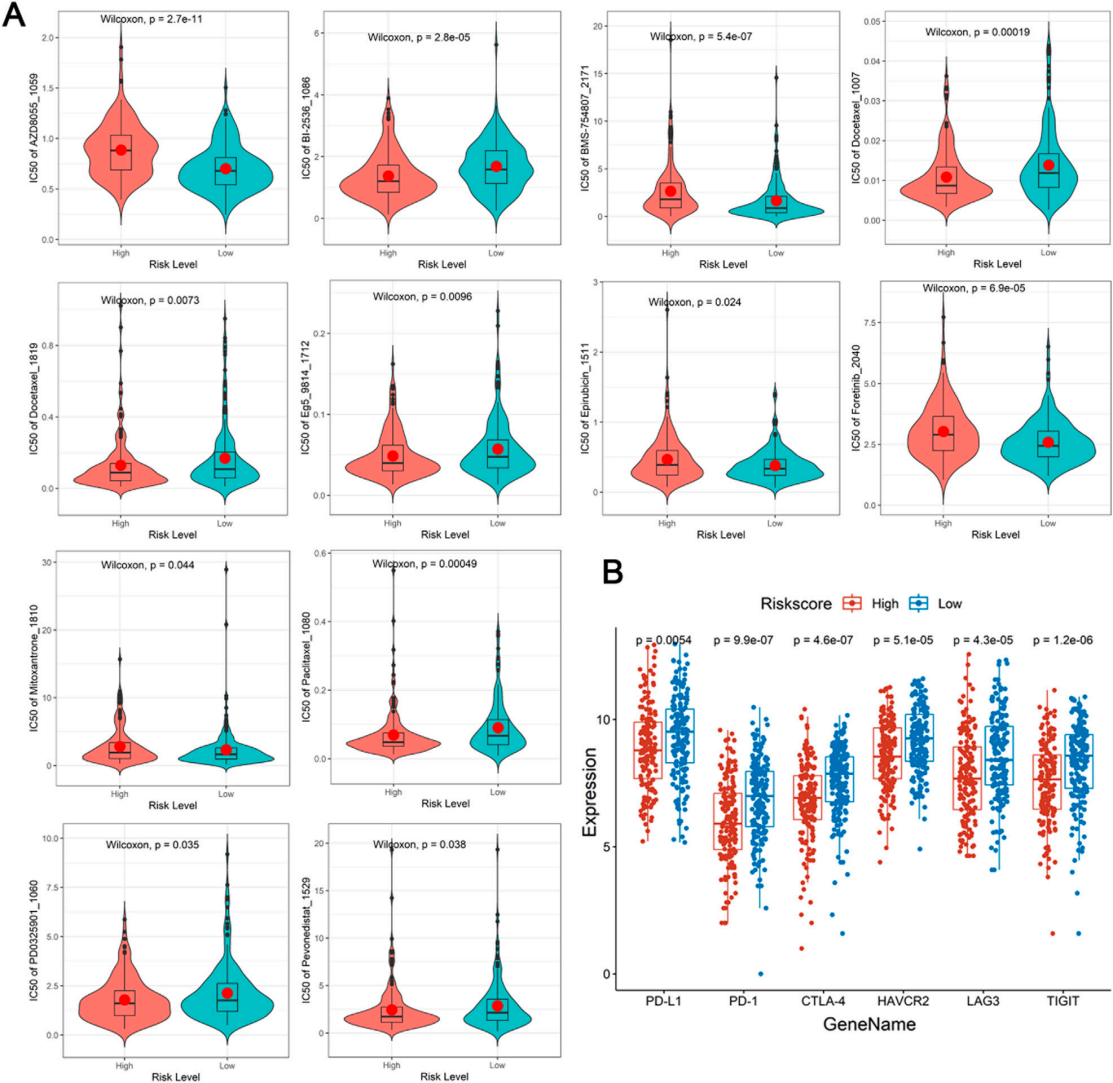
Drugs prediction and immune checkpoints analysis. **(A)** The half-maximal inhibitory concentration (IC_50_) of drugs and small molecule inhibitors between the high- and low-risk group. **(B)** Differences expression in immune checkpoints.

As showed in the violin plots, a sum of 12 drugs showed significant difference between different risk groups, of which 5 upregulated and 7 downregulated in high-risk group (P < 0.05, Figure 6A). Lastly, PD-L1, PD-1, CTLA-4, HAVCR2, LAG3, and TIGIT demonstrated significant difference between different risk groups, notably, they were all high expression in low-risk group (P < 0.05, Figure 6B). Meanwhile, DE-DRLs were generally positively correlated with the majority of the immune checkpoints (Supplementary Table S4).

### 4.6 Verification of DE-DRLs expression

**FIGURE 7:**
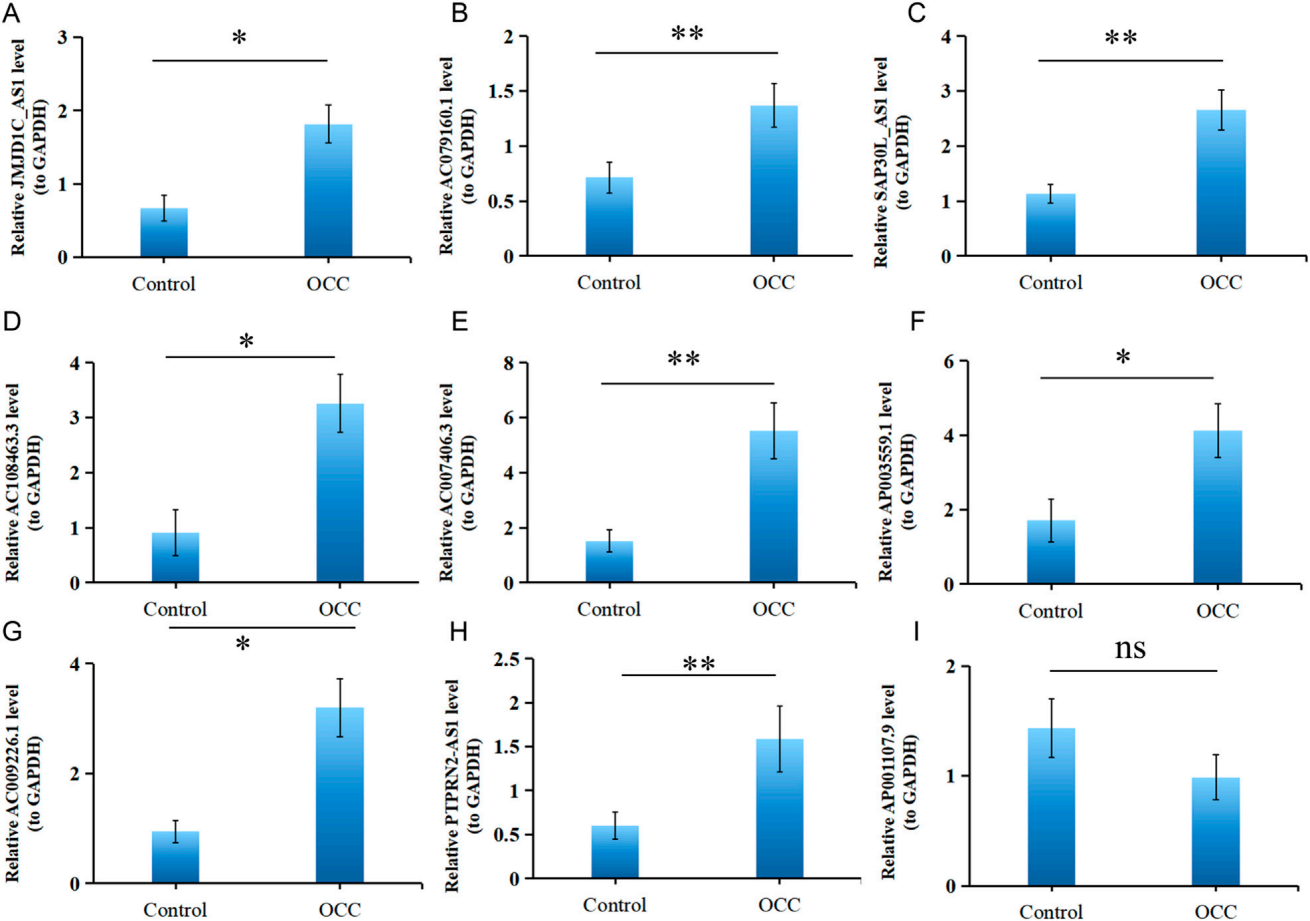
Validation of 9 DE-DRLs. RT-qPCR expression levels of JMJD1C_AS1 **(A)**, AC079160.1 **(B)**, SAP30L_AS1 **(C)**, AC108463.3 **(D)**, AC007406.3 **(E)**, AP003559.1 **(F)**, AC009226.1 **(G)**, PTPRN2_AS1 **(H)**, and AP001107.9 **(I)**. n = 3, “ns” indicates p > 0.05; “*” indicates p < 0.05; “**” indicates p < 0.01; “***” indicates p < 0.001; and “****” indicates p < 0.0001.

Previous studies revealed that 9 DE-DRLs were significantly upregulated in OSCC samples across TCGA-HNSC (P < 0.05) (Figure 2C). These findings prompted further validation of DE-DRLs expression using RT-qPCR. Consistent with the initial results, RT-qPCR demonstrated that AC009226.1, AC108463.3, SAP30L_AS1, AC007406.3, ΑP003559.1, JMJD1C_AS1, JMJD1C_AS1, and AC079160.1 expression were significantly higher (P < 0.05) in OSCC tumor samples compared to control samples (Figure 7). These results further underscored their reliability as biomarkers and highlighted their potential utility in OSCC prognostic diagnosis and therapeutic development.

## 5 Discussion

The OSCC patients face a poor prognosis, regulating disulfidptosis in cancer is a promising therapeutic approach (Yang et al., 2024). In this study, 9 DE-DRLs (AC009226.1, AP001107.9, AC108463.3, SAP30L_AS1, AC007406.3, PTPRN2_AS1, AP003559.1, JMJD1C_AS1, and AC079160.1) associated with OSCC were accurately identified. A prognostic model showed high-risk patients had lower survival. A nomogram integrating risk score and clinical features had excellent predictive ability for patient outcomes. DE-DRLs were linked to the OSCC immune microenvironment, where high levels of certain immune cells predicted poor survival. Additionally, 12 drugs differed in efficacy between risk groups, and immune checkpoints were more highly expressed in the low-risk group, pointing to potential therapeutic targets.

To prevent overfitting, we used LASSO regression to reduce the dimensionality of the data. We identified 9 DE-DRLs that were closely associated with the OS of OSCC patients and all highly expressed in tumor group of TCGA-HNSC. Several of the identified DE-DRLs have been previously characterized and are strongly associated with tumorigenesis and cancer progression. Specifically, AC009226.1 has been established as a prognostic biomarker for lung adenocarcinoma (LUAD), while AC007406.3 has been validated as a prognostic target in clear cell renal cell carcinoma (ccRCC). Both lncRNAs exhibit elevated expression levels in tumor tissues compared to normal counterparts, as reported in previous studies (Sun et al., 2023; Zhang et al., 2023b). In addition, JMJD1C_AS1 and AC079160.1 have been identified as potential prognostic biomarkers for gastric cancer (GC) (Ahmadpour Youshanlui et al., 2024; Guo et al., 2021). Notably, high expression of AC079160.1 has been correlated with favorable survival outcomes in GC patients, suggesting its role in predicting prognosis and potentially informing treatment strategies. These findings underscore the clinical relevance of these DE-DRLs across diverse cancer types and highlight their potential utility in precision oncology (Guo et al., 2021; Yang et al., 2022). Moreover, emerging evidence demonstrates that SAP30L-AS1 exhibits elevated expression levels in both prostate cancer (PCa) tissues and cell lines. Functionally, SAP30L-AS1 suppresses the upregulation of SAP30L in PCa, thereby exerting a tumorigenic effect. These findings, as previously reported (Qin et al., 2019), further highlighting its broad clinical relevance. PTPRN2_AS1 displayed consistent result with Xiong et al. (2023). Notably, this study represents the first report identifying AC108463.3 and AP003559.1 as prognostic markers for cancer, thereby addressing a critical void in the existing cancer prognosis literature. This groundbreaking discovery significantly expands the current knowledge base regarding potential cancer biomarkers. RT-qPCR analysis revealed elevated expression levels of AP001107.9 in the control group, diverging from the findings of prior investigations by Shen et al. (2025). This discrepancy may stem from two main factors: limited sample size causing heterogeneity and study-specific epigenetic/post-transcriptional regulation of AP001107.9. Uncharacterized regulatory mechanisms in prior research may explain expression variability and result divergence. Exploring DE-DRLs-prognosis correlations could offer new molecular targets for OSCC.

Systematically analyzing the biological pathways that lncRNAs may be involved in regulating will help us deepen our understanding of the pathological process of OSCC from multiple dimensions, from molecular mechanisms to clinical applications, providing dual support for target selection and efficacy prediction in the precise treatment of OSCC. Previous studies have shown that, the downregulation of SAP30L by SAP30L_AS1 impairs the assembly and enzymatic activity of the HDAC complex. This leads to dysregulated histone deacetylation, subsequent transcriptional repression of tumor-suppressor genes, and promotion of cancer cell proliferation (Qin et al., 2019). PTPRN2_AS1 can adsorb miR-145-5p through the ceRNA mechanism (Huang et al., 2021), thereby relieving its inhibition on PTPRN2 and enhancing the activity of the insulin signaling pathway, promoting tumor cell glycolysis (Meng et al., 2019), which also crucial in the energy metabolism reprogramming of OSCC. However, although no direct studies have confirmed the involvement of AC009226.1, AP001107.9, AC108463.3, AC007406.3, AP003559.1, JMJD1C_AS1, and AC079160.1 in the physiological processes of OSCC, evidence from the literature and the present study indicates that these lncRNAs may participate in biological pathways related to tumor proliferation (Cutilli et al., 2016), invasion (Wu et al., 2022), immune regulation (Huang et al., 2024), and epigenetics (Tang et al., 2021). DNA methylation is one of the most important epigenetic mechanisms to regulate gene expression, aberrant DNA methylation patterns are strongly associated with cancer, such as lncRNAs can recruit or repel DNA modifiers to specific gene targets and regulate the expression of DNA modifiers *per se* at multiple levels (Huang et al., 2022; Shen et al., 2017). This not only provides novel insights into the multi-dimensional regulatory mechanisms underlying OSCC progression but also lays a foundation for identifying potential prognostic biomarkers and therapeutic targets for OSCC.

While the OSCC pathogenesis also involves complex host-microenvironment interactions, and clinical challenges of advanced OSCC further highlight the need to expand lncRNA-related mechanism exploration to broader pathological and clinical contexts. Furthermore, previous studies have shown that the ecological imbalance of the oral microbiome caused by risk factors such as tobacco use, alcohol consumption, betel nut chewing, and HPV infections can lead to abnormal expression of lncRNAs in the oral cavity, which are associated with inflammatory mechanisms facilitating OSCC progression (Crispino et al., 2024; Mishra et al., 2025). More importantly, since OSCC patients were usually diagnosed at an advanced stage and are at risk of bone metastasis, which indicate a poor outcome (Wang et al., 2024). Nevertheless, there has been limited information on bone metastasis in OSCC (Wang et al., 2024). According to previous research results, inhibition of disulfidptosis can prevent osteoclast overactivation and, consequently, reduce the risk of bone metastasis in patients (Clézardin et al., 2021). This indicates that targeting disulfidptosis-related lncRNAs is expected to regulate the balance of intracellular disulfidptosis bond metabolism and the activity of osteoclasts at the molecular level, thereby blocking the colonization and invasion of tumor cells into bone tissue. This reminds us that by targeting the disulfidptosis-related lncRNAs screened in this study, we can not only inhibit the progression of the primary lesion of OSCC but also reduce the risk of bone metastasis, providing a new therapeutic target for improving the quality of life of patients with advanced OSCC.

At present, incorporating prognostic models into routine clinical practice can facilitate the estimation and quantification of patient prognosis (Hoesseini et al., 2022). The AUCs of prognostic genes for the OSCC by Li et al. at 1-year was 0.683 (Li et al., 2024) and Tang et al. was 0.752 (Tang et al., 2023), while our study result was 0.754. The nomogram represents a key medical advancement, addressing personalized healthcare needs by enabling clinicians to evaluate individual risk factors and prognosis for informed, tailored decisions (Balachandran et al., 2015), the slope of the calibration curve in this study was approximately equal to 1. This study validates the prognostic model and nomogram for accurately predicting OSCC outcomes. With high precision, these tools enhance risk stratification and evidence-based treatment decisions, improving patient management and personalized care.

Accumulating evidence from prior investigations has firmly established the indispensable role of immune cell infiltration in shaping the therapeutic response and clinical outcomes of patients with OSCC. In the present study, mast cells, activated NK cells, and Tregs emerged as key immune cell populations with significant implications for OSCC immunobiology and prognosis. This observation aligns with established literature demonstrating that tumor-infiltrating mast cells can modulate Treg activity to facilitate tumor progression, thereby undermining the host’s antitumor immune response (Lv et al., 2024). Consistently, elevated densities of activated NK cells and Tregs within the tumor microenvironment have been repeatedly associated with adverse clinical outcomes in OSCC patients (Lv et al., 2024; Wu et al., 2020). Our findings also corroborate previous reports indicating that a high TMB in OSCC patients portends a poorer prognosis and serves as a valuable biomarker for guiding treatment decisions in recurrent and metastatic disease (Lv et al., 2024). Notably, differential expression analysis revealed significantly higher levels of MYCBP2, TGFBR2, PLEC, and NSB1 in the high-risk cohort, suggesting potential roles for these genes in tumorigenesis and disease progression. In thyroid cancer (TC), MYC binding protein 2 (MYCBP2) has been linked to inflammatory cell infiltration and patient survival (Wang et al., 2022). TGFBR2, a central transducer of TGF-β - mediated growth inhibitory signals, has been implicated in the pathogenesis of multiple malignancies (Huang et al., 2014). PLEC has been identified as a promising biomarker and therapeutic target in pancreatic adenocarcinoma (PAAD) (Ge et al., 2024), while amplification of NSB1 has been shown to drive the pathobiology of uveal melanoma (UM) (Banimohammad et al., 2025). Collectively, these findings prompt the hypothesis that disulfidptosis may represent a critical mechanism underlying immune resistance and tumor immune escape in OSCC, offering novel insights into the complex interplay between cell death pathways and tumor immunobiology.

In recent years, cancer bioinformatics has emerged as a transformative force, driving advancements in anticancer drug development and personalized therapeutics while substantially enhancing the accuracy of cancer therapeutic prediction (Lu et al., 2017). Treatment resistance, a common occurrence in OSCC, substantially diminishes patient survival rates and profoundly impacts clinical treatment decisions (Cheng et al., 2021). Consequently, a comprehensive assessment of drug sensitivity in OSCC patients is imperative. In the present study, five drugs, including BMS-754807_2171, AZD8055_1059, Epirubicin_1511, Mitoxantrone_1810, and Foretinib_2040, were identified as upregulated in the high-risk group. These findings may help elucidate the mechanisms underlying the poor prognosis observed in this subgroup. Previous research has shown that BMS-754807 potentiates the efficacy of chemotherapeutic agents in lung cancer cells by inducing autophagy, cell cycle arrest, and growth inhibition, ultimately leading to synergistic cytotoxicity (Zhang and Zhou, 2025). Similarly, Yin et al. (2024) reported consistent IC_50_ expression of BMS-754807 in gastric adenocarcinoma (STAD) in their study on lactylation-related gene sets and mitochondrial functions in STAD (Yin et al., 2024). AZD8055 is a potent, selective, and orally bioavailable ATP-competitive mammalian target of rapamycin kinase inhibitor with *in vitro* and *in vivo* antitumor activity (Chresta et al., 2010), AZD8055_1059 had been verified as a drug treatment target in non-small cell lung cancer (Shi et al., 2025). While, the Epirubicin was already used in clinical research for non-small cell lung cancer as early as 1997 by Colucci et al. (1997). Furthermore, Foretinib, a multikinase inhibitor, has been clinically employed in the treatment of breast carcinoma (BC) (Anitha et al., 2023) and Mitoxantrone_1810 had also been proven to have a higher expression level in the high-risk group of HNSCC (Zhu et al., 2022). Collectively, the results of this study provide a theoretical foundation for the development of personalized treatment strategies for OSCC patients.

## 6 Conclusion

In the current investigation, transcriptomic data integrated with advanced bioinformatics methodologies were utilized to systematically identify 9DE-DRGs, namely, AC009226.1, AP001107.9, AC108463.3, SAP30L_AS1, AC007406.3, PTPRN2_AS1, AP003559.1, JMJD1C_AS1, and AC079160.1, as differentially expressed in OSCC. A prognostic model and nomogram showed strong predictive value for patient outcomes, aiding clinical risk stratification. Immune microenvironment analysis revealed interactions between cell death pathways and tumor immunobiology. Drug prediction identified BMS-754807_2171 and Foretinib_2040 as potential therapies. Limitations include data quality, algorithm assumptions, and reliance on RT-qPCR validation, necessitating independent clinical validation across cohorts to confirm biomarkers utility in OSCC management.

## Data Availability

All datasets used in this study are publicly available on the UCSC Xena platform in cohort: GDC TCGA Head and Neck Cancer (HNSC), available at: https://xenabrowser.net/datapages/.

## Abbreviations

OSCC, Oral squamous cell carcinoma; lncRNAs, long non-coding RNAs; DRLs, disulfidptosis-related lncRNAs; DRGs, disulfidptosis-related genes; DE-DRLs, differentially expressed DRLs; RT-qPCR, reverse transcription-quantitative polymerase chain reaction; CI, confidence intervals; NK, natural killer; OS, overall survival; TMB, tumor mutational burden; TCGA, the Cancer Genome Atlas; ROC, receiver operating characteristic; AUC, area under curve; IC_50_, half maximal inhibitory concentration; LUAD, lung adenocarcinoma; ccRCC, clear cell renal cell carcinoma; GC, gastric cancer; PCa, prostate cancer; HNSCC, head and neck squamous cell carcinoma; TC, thyroid cancer; MYCBP2, MYC binding protein 2; PAAD, pancreatic adenocarcinoma; UM, uveal melanoma; STAD, gastric adenocarcinoma; BC, breast carcinoma.
