# Supplementary material for: Unveiling disulfidptosis-linked lncRNA signatures: insights into the immune microenvironment and drug responsiveness in oral squamous cell carcinoma
Source: Front Genet. 2025 Nov 10;16:1650544. doi: 10.3389/fgene.2025.1650544 (PMC12640761; doi:10.3389/fgene.2025.1650544)
Supplement: Supplementary file 2 [file Table1.docx]

**Supplementary Table 1 Clinical pathological information of 337 OSCC samples**

|  | **Alive** | **Death** | **Overall** |
| --- | --- | --- | --- |
|  | **(N=185)** | **(N=152)** | **(N=337)** |
| factor(Gender) |  |  |  |
| female | 50 (27.0%) | 53 (34.9%) | 103 (30.6%) |
| male | 135 (73.0%) | 99 (65.1%) | 234 (69.4%) |
| factor(Age) |  |  |  |
| <=60 | 97 (52.4%) | 65 (42.8%) | 162 (48.1%) |
| >60 | 88 (47.6%) | 87 (57.2%) | 175 (51.9%) |
| factor(Stage) |  |  |  |
| I | 16 (8.6%) | 3 (2.0%) | 19 (5.6%) |
| II | 35 (18.9%) | 20 (13.2%) | 55 (16.3%) |
| III | 37 (20.0%) | 23 (15.1%) | 60 (17.8%) |
| IV | 78 (42.2%) | 90 (59.2%) | 168 (49.9%) |
| Missing | 19 (10.3%) | 16 (10.5%) | 35 (10.4%) |
| factor(T) |  |  |  |
| T0 | 1 (0.5%) | 0 (0%) | 1 (0.3%) |
| T1 | 25 (13.5%) | 6 (3.9%) | 31 (9.2%) |
| T2 | 65 (35.1%) | 37 (24.3%) | 102 (30.3%) |
| T3 | 26 (14.1%) | 39 (25.7%) | 65 (19.3%) |
| T4 | 52 (28.1%) | 59 (38.8%) | 111 (32.9%) |
| Missing | 16 (8.6%) | 11 (7.2%) | 27 (8.0%) |
| factor(N) |  |  |  |
| N0 | 80 (43.2%) | 41 (27.0%) | 121 (35.9%) |
| N1 | 36 (19.5%) | 13 (8.6%) | 49 (14.5%) |
| N2 | 43 (23.2%) | 65 (42.8%) | 108 (32.0%) |
| N3 | 1 (0.5%) | 2 (1.3%) | 3 (0.9%) |
| Missing | 25 (13.5%) | 31 (20.4%) | 56 (16.6%) |
| factor(M) |  |  |  |
| M0 | 80 (43.2%) | 42 (27.6%) | 122 (36.2%) |
| MX | 27 (14.6%) | 15 (9.9%) | 42 (12.5%) |
| Missing | 78 (42.2%) | 95 (62.5%) | 173 (51.3%) |
