# Supplementary material for: Unveiling disulfidptosis-linked lncRNA signatures: insights into the immune microenvironment and drug responsiveness in oral squamous cell carcinoma
Source: Front Genet. 2025 Nov 10;16:1650544. doi: 10.3389/fgene.2025.1650544 (PMC12640761; doi:10.3389/fgene.2025.1650544)
Supplement: Supplementary file 3 [file Table2.docx]

| JMJD1C_AS1-F | CAGGTGAGGGGAAAGTGAAGTGT |
| --- | --- |
| JMJD1C_AS1-R | GTGTTGTCCTGCTCTGCTGCT |
| AC079160.1-F | CCCTATTTGCCTGGGTATCAC |
| AC079160.1-R | GGCATCTGGCAGGTGACT |
| lnc SAP30L_AS1-F | AGTGCAACGTCCAGGCATTT |
| lnc SAP30L_AS1-R | GGATGGCAGGTTTTCCCTTC |
| AC108463.3-F | AGAGCAGAAGACAAAGCCGA |
| AC108463.3-R | CATGCCTGGCTGAAGGAGAG |
| AC007406.3-F | GAGAGCACTCCGCCCA |
| AC007406.3-R | GTTAAATGAGGGCGCAGGAAT |
| ΑP003559.1-F | TGGGATTCGTCCAGCATCAT |
| ΑP003559.1-R | GCAACTAGGGGGACATGAGAC |
| GAPDH-F | CGCTCTCTGCTCCTCCTGTTC |
| GAPDH-R | ATCCGTTGACTCCGACC |
| AC009226.1-F | CGCCCATGACTCACTTCTCTT |
| AC009226.1-R | TACCATCGCTGTCTTCTCCCT |
| AP001107.9-F | TCCTGCCTATTTGGAGCAGC |
| AP001107.9-R | CATCCCTCGTGCCTAACACA |
| PTPRN2-AS1-F | AGGCCCTGAGAAAGAACAGAAA |
| PTPRN2-AS1-R | CGGTGGTTCCACTCTCAAGG |

**Supplementary Table 2 Primers sequence of GAPDH and DE-DRLs**
